# *Candida* and Complement: New Aspects in an Old Battle

**DOI:** 10.3389/fimmu.2020.01471

**Published:** 2020-07-14

**Authors:** Verena Harpf, Günter Rambach, Reinhard Würzner, Cornelia Lass-Flörl, Cornelia Speth

**Affiliations:** Institute of Hygiene and Medical Microbiology, Medical University of Innsbruck, Innsbruck, Austria

**Keywords:** *Candida*, complement, evasion, innate immunity, factor H, invasive fungal infections

## Abstract

*Candida* is a dominant fungal pathogen in immunocompromised hosts, leading to opportunistic infections. Complement with its multifaceted functions is involved in the immune defense against this yeast, and recently several novel aspects have emerged in this old battle. It is clear that *Candida* can adopt both roles as a colonizer or as a pathogen. In our article, we focus on the molecular mechanisms of the *Candida*-complement interplay, which occur in disseminated disease as well as locally on skin or on mucous membranes in mouth and vagina; the mechanisms can be supposed to be the same. Activation of the complement system by *Candida* is facilitated by directly triggering the three dominant pathways, but also indirectly via the coagulation and fibrinolysis systems. The complement-mediated anti-*Candida* effects induced thereby clearly extend chemotaxis, opsonization, and phagocytosis, and even the membrane attack complex formed on the fungal surface plays a modulatory role, although lysis of the yeast *per se* cannot be induced due to the thick fungal cell wall. In order to avoid the hostile action of complement, several evasion mechanisms have evolved during co-evolution, comprising the avoidance of recognition, and destruction. The latter comes in many flavors, in particular the cleavage of complement proteins by yeast enzymes and the exploitation of regulatory proteins by recruiting them on the cell wall, such as factor H. The rationale behind that is that the fluid phase regulators on the fungal cell surface down-regulate complement locally. Interestingly, however, evasion protein knockout strains do not necessarily lead to an attenuated disease, so it is likely more complex *in vivo* than initially thought. The interactions between complement and non-*albicans* species also deserve attention, especially *Candida auris*, a recently identified drug-resistant species of medical importance. This is in particular worth investigating, as deciphering of these interactions may lead to alternative anti-fungal therapies directly targeting the molecular mechanisms.

## The Complement System and Its Multifaceted Functions

The complement system is an ancient and effective multicomponent bodily system for antimicrobial defense, homeostasis, and immunomodulation [reviewed in (1, 2)]. These functions are elicited by a system of soluble and membrane-bound effector, regulator, and receptor molecules, and form a tightly regulated cascade. Complement detects invading microbes and modified self-surfaces (e.g., on apoptotic cells) using various pattern recognition molecules. Via different effector molecules of the cascade, this detection results in opsonization with subsequent phagocytic clearance, formation of the lytic C5b-9 complex of the terminal pathway, induction of inflammation, and modulation of other innate, and adaptive immune weapons.

Three activation pathways can push this system: the classical pathway (mainly induced by binding of immunoglobulins on target surfaces), the alternative pathway (triggered by deposition of spontaneously formed C3b on foreign surfaces with subsequent amplification loop), and the lectin pathway (elicited by foreign or aberrant carbohydrate structures on surfaces). The consequence of all three pathways is the covalent deposition of complement on the foreign surface and the generation of a C3-convertase that cleaves complement factor C3 into the larger C3b fragment and the smaller C3a anaphylatoxin. The composition of the C3-convertase varies upon the activation pathway (C4b2b for the classical and lectin pathway and C3bBb for the alternative pathway). The newly formed C3b either can amplify the alternative pathway by generating new C3-convertase molecules or can form a complex with the existing C3-convertases to generate a C5-convertase. The enzymatic function of the C5-convertases (cleavage of complement protein C5 into C5a anaphylatoxin and the C5b fragment) initiates the sequence of the terminal pathway. Several aggregation events finally result in generation of the C5b-9 complex. Inserted into the target membrane, C5b-9 is called membrane attack complex (MAC) and induces lysis of the pathogen or altered cell (3). If soluble, C5b-9 (called terminal complement complex TCC) harbors further functions such as cell activation and pro-inflammatory immune activation (4, 5).

**Figure 1:**
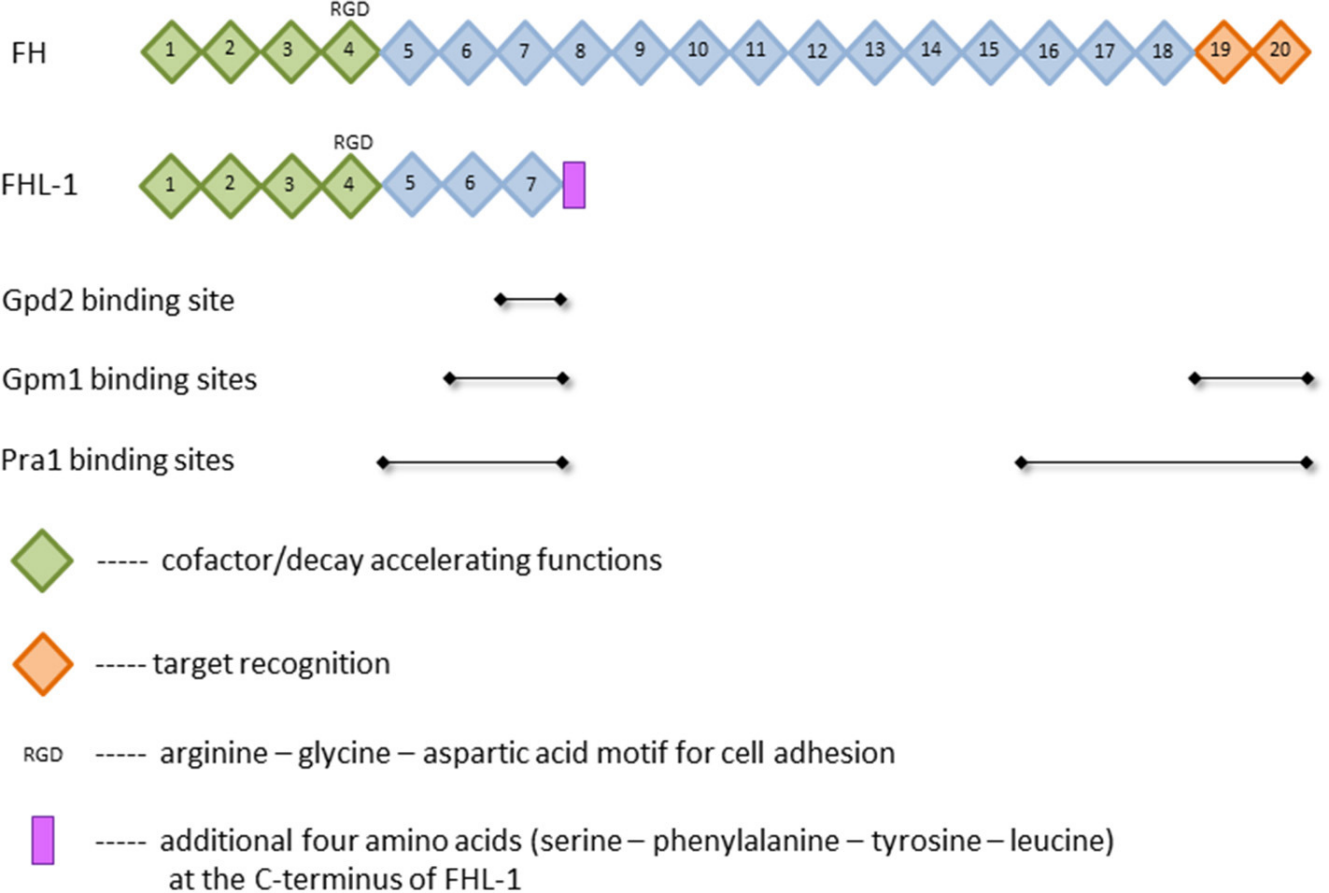
Structure of factor H (FH) and factor H-like protein 1 (FHL-1). Depicted are selected functions of short consensus repeats (SCR), illustrated as squares, and the known binding sites of the FH-binding proteins phosphoglycerate mutase 1 (Gpm1), pH-regulated antigen 1 (Pra1), and glycerol-3-phosphate dehydrogenase 2 (Gpd2). For further details and references, see text.

The presence of such a powerful tool in the body requires the tight control by numerous regulators. Major soluble controller of the alternative pathway is factor H (FH), which is a single chained 150 kDa plasma glycoprotein (6). This protein is composed of 20 homologous domains known as short consensus repeats (SCR) or complement control protein (CCP) modules, each of which is made of 60 amino acids and stabilized by two internal disulfide-bonds (7, 8). The SCRs bear different functions. While the C-terminal domains SCRs 19 and 20 are essential for the target recognition, the N-terminal domains SCRs 1–4 mediate the diverse complement regulator functions (9–11) (Figure 1). Complement regulation by FH can be divided into three parts: First, FH competes with factor B for the C3b binding and therefore inhibits the assembly of the alternative pathway C3- and C5-convertases. Second, the protein exhibits a “decay accelerating activity,” as it enhances the disassembly of these convertases by displacing bound factor Bb; and third, FH is used as a cofactor for the serine protease factor I in cleaving and inactivating C3b (8, 12, 13). An alternatively spliced transcript of the FH gene is factor H-like protein 1 (FHL-1) (14, 15). This 43 kDa plasma glycoprotein consists of the N-terminal domains SCRs 1–7 with four additional amino acids added at the C-terminus (16) (Figure 1). Compared to FH, the truncated form FHL-1 is less abundant in plasma (500 vs. 50 μg/ml, respectively) (17). FHL-1 still bears the same complement regulatory functions as FH and is therefore an important protein of the alternative pathway (8, 12, 13). The classical and lectin pathway, however, are mainly controlled by C4b binding protein (C4BP) [reviewed in (18)]. It binds to and inhibits the function of the activated complement compound C4b. C4BP acts as a cofactor of factor I (FI)-mediated cleavage and inactivation of soluble and cell-bound C4b, thus preventing the assembly of the C3-convertase C4b2b. In addition, C4BP accelerates the decay of functional C4b2b. Although FH is the main controller of the alternative pathway, C4BP also affects this pathway since it acts as a cofactor of FI in the proteolytic cleavage of C3b (18, 19).

A similar cofactor activity is attributed to the cell surface-expressed regulator molecule membrane cofactor protein (MCP; CD46). MCP and a further surface regulator, decay accelerating factor (DAF, CD55), accelerate the decay of both C3 convertases (3). Important regulators of the terminal pathway are CD59 (protectin) and vitronectin. Both molecules interfere with assembly of the C5b-9 complex and thus prevent its insertion into the membrane to form a lytic pore (20, 21).

## *Candida* as Colonizer and Pathogen

*Candida spp*. are common members of the human mycobiome, but also opportunistic fungal pathogens [reviewed in (22, 23)]. In healthy individuals, *Candida* resides on the skin or as colonizer of the oral cavity, the gastrointestinal and the urogenital tract. When shifting from a colonizer to a pathogen, it causes cutaneous and mucocutaneous candidiasis as well as life-threatening invasive infections of inner organs and the bloodstream. The shift is enabled by changes in the host microbiota (e.g., by antibiotics), impairment of the host immune response (e.g., by immunosuppressive therapy), or alterations in the local conditions (e.g., shifts in nutrients or pH) (23, 24). The most common manifestation is vulvovaginal candidiasis affecting millions of women worldwide (25). The growing number of immunocompromised patients with intravenous catheters, cancer chemotherapy, or organ transplantations contributes to the boost in *Candida*-induced bloodstream infections (26).

The genus *Candida* includes at least 30 species of clinical importance; the most frequent one is *Candida albicans*, but non-*albicans* species become more and more common and are often associated with reduced antifungal susceptibility and outbreaks (22). *Candida auris* is a recently discovered emerging multidrug-resistant species that is responsible for an increasing number of nosocomial outbreaks.

Understanding of anti-*Candida* host defense mechanisms remains an urgent need. One focus is the interaction with the complement system as a universal and fast-acting immune weapon. The relevance of this interaction is accentuated by the fact that local production of complement is revealed for most organs, indicating a role in both superficial candidiasis and in *Candida* sepsis. The picture of *Candida*—complement interplay is complex and multifaceted and will be depicted in the following. Most knowledge exists about *Candida albicans*, but also non-*albicans* species are included, particularly *Candida auris*.

## *Candida* spp. Trigger Complement Activation by Multiple Pathways

Various pathways are capable to mediate complement activation by *Candida*, underlining the relevance of this innate immune system in the case of infection.

The direct mechanisms of complement activation include the classical, lectin, and alternative pathway, which all were described to be triggered by *Candida* (27–29). Early experiments already showed that classical and alternative pathway are activated with different kinetics (30). The classical pathway might not only be triggered by interaction between the pattern recognition molecule C1q and specific anti-*Candida* antibodies: a recent report described that serum amyloid P (SAP) component, a member of the pentraxin family, binds to the *Candida albicans* surface (31). Although this report shows reduced phagocytosis after SAP binding, other authors described that SAP is able to activate the classical complement pathway (32, 33).

The efficiency of complement to react on the presence of *Candida* strongly depends on the surface composition of the yeast. On intact *Candida albicans* cells, β-glucan is located, together with chitin, in the inner fungal cell wall. However, during C. *albicans* infection or by treatment with caspofungin, the inner β-glucan components become exposed (34) and can initiate the alternative pathway of the complement system (35). Similarly, purified β-glucan triggered the alternative pathway when co-incubated with the purified AP proteins (35).

The lectin pathway is an evident candidate for *Candida*-triggered complement activation, since mannans represent about 40% of the total polysaccharide yeast cell wall content (36). Neth et al. (29) found diverse *Candida* species to strongly bind MBL (mannan-binding lectin), a starter molecule of the lectin pathway, with subsequent C4 deposition on the yeast surface. Another pattern recognition molecule of the lectin pathway, collectin-11, also bound to carbohydrate residues on *Candida albicans* and triggered complement activation with C4b and C3b deposition on the surface (37). Blocking of MBL in a mouse model increased C. *albicans* colonization, and MBL-deficient animals had a higher level of colonization than wild-type mice (38).

**Figure 2:**
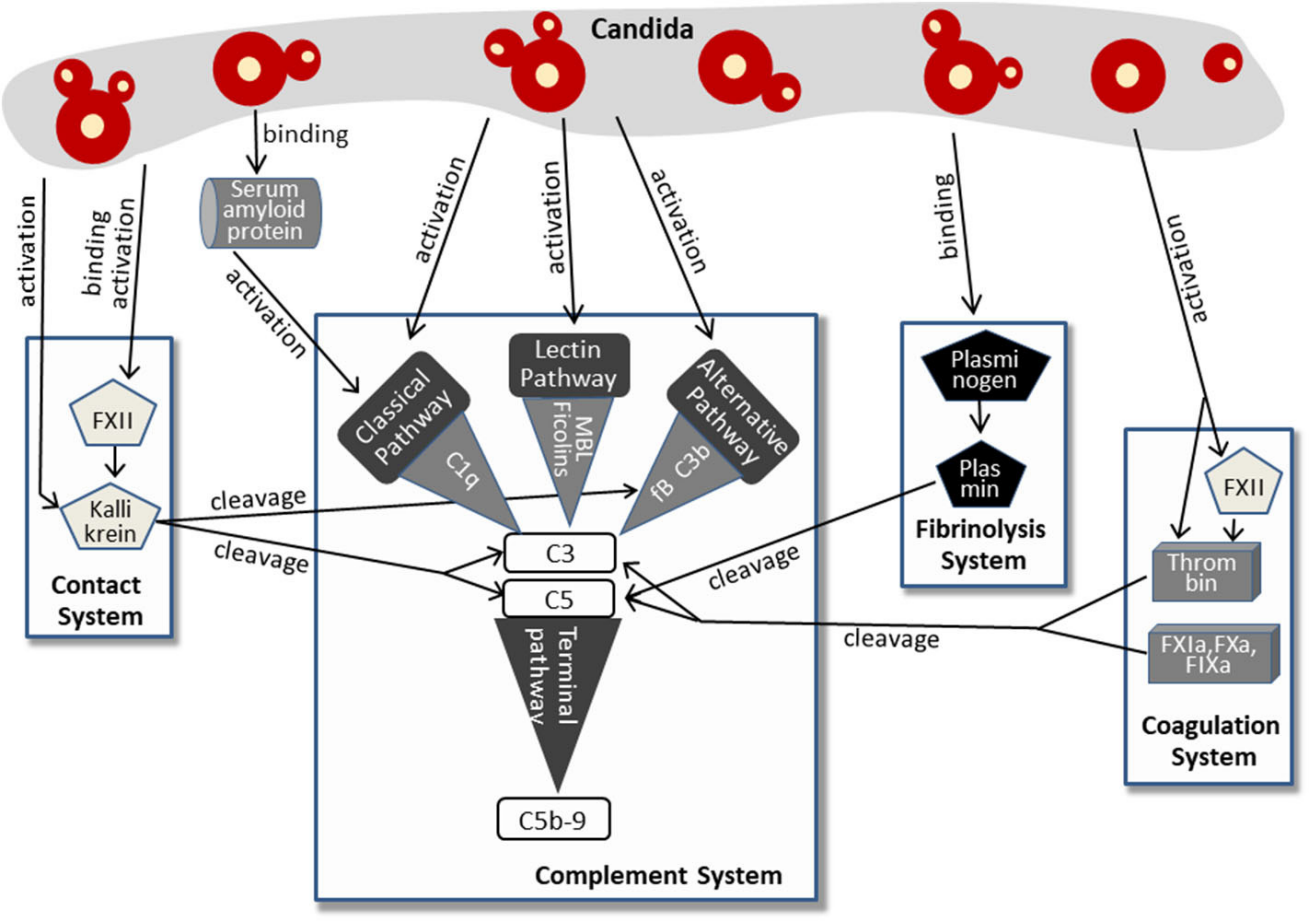
Pathways of complement activation by *Candida*, either directly or by affecting the contact system, fibrinolysis system, or coagulation system. For further details, see text.

More recent findings imply that *Candida* activates the complement system not only via classical, lectin, or alternative pathway. More and more indirect mechanisms were discovered, involving molecules of the contact system, the fibrinolysis system, and the coagulation system. This complex pattern of *Candida*-induced complement activation is summarized in Figure 2.

The human plasma contact system represents a powerful immune surveillance tool that is activated by negatively charged surfaces, (e.g., on fungi). The contact of factor XII (FXII) to these surfaces triggers autoactivation with subsequent conversion to the active serine protease FXIIa. This process is also induced by contact with the cell walls of *Candida albicans* and *C. tropicalis* (39). In addition, previous work revealed that *Candida*-derived proteinases also activate factor XII (40), which appears to be a multifunctional molecule in innate immunity (41). Active FXIIa, either generated by *Candida* surface contact or by *Candida* proteases, cleaves prekallikrein to form kallikrein. Kallikrein reciprocally activates FXIIa, thus increasing rapidly the plasma level of both enzymes (41, 42). Furthermore, a *Candida albicans* proteinase directly converts plasma prekallikrein to active kallikrein (43). Kallikrein was shown to cleave the central complement protein C3 to yield the active fragments C3b and C3a. The proteolytic cleavage site used by kallikrein corresponds to that used by the C3 convertase, and the kallikrein-generated fragment C3b forms C3-convertase, thus triggering the C3 amplification loop. In addition, the C3a fragment resulting from kallikrein cleavage was determined to be biologically active and to harbor antimicrobial activity (44). The link from the contact system to complement activation in *Candida* infection is strengthened by findings that kallikrein also cleaves factor B, yielding Bb and Ba and thereby triggering the alternative pathway (44). Furthermore, contact system and complement system share regulator mechanisms, since C1INH, the regulator of the proteases of all three complement pathways, also represents the primary plasma control for FXIIa and kallikrein [reviewed in (45, 46)].

A second system that is activated by *Candida* and that cross-triggers complement activation is the coagulation system (Figure 2), a complex set of enzymes that mediates blood clotting. FXII, described above to be activated by *Candida* surfaces or *Candida* proteinases, also plays a central role in this system and promotes the generation of active thrombin (47), a key enzyme of coagulation (48). Active thrombin also results directly from the enzymatic function of *Candida* proteases (40). Thrombin effectively cleaves C3 to generate C3a that was capable to induce a chemotactic response of neutrophils (49, 50). In addition, thrombin is able to cleave C5 and thereby generates biologically active C5a and a functional C5-convertase even in the absence of C3 (51). Thrombin-generated C5 products also support triggering of the terminal complement pathway (52). Other components of the coagulation system, the factors IXa, Xa, and XIa, were also capable to cleave C3 and C5 (49).

The third system triggered by *Candida* and contributing to *Candida*-induced complement activation is the fibrinolysis system (Figure 2). Here, plasminogen/plasmin represent the central molecules. Urokinase-type plasminogen activator cleaves the plasma protein plasminogen to form plasmin, a serine protease that lyses fibrin clots and promotes degradation of the extracellular matrix. *Candida albicans* binds plasminogen to its surface via the proteins Gpm1 and Pra1 (53, 54). After conversion into proteolytically active plasmin, the subsequent degradation of extracellular matrix proteins and cell junction proteins might favor dissemination of the yeast. However, plasmin also mediates complement activation by cleaving C3 and C5 (49), and the plasmin-activated C5 was capable to yield a functional membrane attack complex (MAC) (55, 56). It must be mentioned that the role of plasminogen/plasmin in complement activation seems to be ambiguous, since other reports also describe an inhibitory role and list plasminogen as a complement inhibitor (see below).

## Complement-Mediated Anti-*Candida* Effects

The infection-induced complement activation leads in general to opsonization and lysis of invading pathogens, initiation of inflammation, guidance of immune cells to the site of infection, and last but not least to stimulation of the adaptive immune response (57). The central role of complement in the host defense against *Candida* infection has been clearly demonstrated in a variety of experimental models with various deficiencies for complement proteins. C3-depleted guinea pigs showed higher susceptibility to lethal *Candida* infections than untreated control animals (58). Tsoni et al. revealed a higher susceptibility to develop systemic and lethal infections with *C. albicans* and *C. glabrata* in C3 deficient mice compared to immunocompetent animals (59). Mice congenitally deficient in C5, such as DBA/2 or A/J, were highly susceptible to C. *albicans* infection (60–62). Furthermore, mice deficient in MBL, or double knockout mice lacking factor B and C2, showed highly increased mortality and elevated fungal load in the tissues after infection with *C. albicans* (63). The central role of complement in *Candida* infection has also been investigated in vaginal candidiasis. Notably, MBL as pattern recognition molecule plays a predominant role to determine severity and recurrence of disease. MBL was demonstrated to bind to *Candida* colonizing the vaginal cavity (64). The gene coding for MBL protein is polymorphic. A polymorphism associated with unstable MBL protein and thus decreased MBL concentrations is more prevalent in women developing primary vulvar vestibulitis syndrome than in control groups (65). Other reports confirm the relation of MBL polymorphism with recurrent vulvovaginal candidiasis and therapy resistance (36, 66–68). Similarly, cutaneous candidiasis was described to be strongly associated with complement. Mice with deficiency in C5 were less efficient in clearing experimental cutaneous *Candida* infection (69). Complement activation and deposition of C3 and factor B on *Candida* surface was demonstrated in an *in vitro* model of cutaneous candidiasis and were suggested to inhibit *Candida* adherence to corneocytes (70).

One predominant role of complement factor C3 is the opsonization of foreign structures to facilitate their uptake by complement receptor-bearing phagocytes (71). Opsonization of the *Candida* surface with C3 fragments is a very fast and efficient process, underlining the relevance of this mechanism particularly in early stages of infection (28). The most prominent receptor for C3b and iC3b deposited on the *Candida* surface is complement receptor 3 (CR3), widely expressed in professional phagocytes. Interestingly, *Candida* exposes a CR3-like molecule on its surface (72) that binds the negative complement regulator factor H and thus contributes to complement evasion, as described below.

Phagocytosis of *Candida* is not only facilitated by deposition of C3 fragments, but is further supported by an MBL-mediated “shortcut.” Besides being the main pattern recognition molecule of the lectin pathway, MBL was shown to possess opsonic functions itself to bridge the *Candida* surface to the cellular complement receptor CR1 (CD35) expressed on monocytes and neutrophils (73). MBL-mediated opsonophagocytosis of *Candida albicans* by neutrophils is independent from complement activation and stimulates the intracellular expression of Dectin-1 with subsequent production of reactive oxygen species (ROS) (74). Since generation of toxic ROS represents an important fungicidal mechanism employed by innate immune cells, MBL acts as a dual complement effector molecule that utilizes both complement activation and activation-independent techniques for antifungal protection.

The pro-inflammatory anaphylatoxins C3a and C5a, which are generated by cleavage of complement factors C3 and C5, support these anti-*Candida* effects by chemotactically attracting phagocytes to the site of infection (75). C5a is particularly potent to attract neutrophils and monocytes; by binding to the corresponding receptors on neutrophils, C5a also increases their antimicrobial effector functions toward *Candida albicans* such as phagocytosis, oxidative burst, and degranulation (76). The induction of the release of the pro-inflammatory cytokines IL-6 and IL-1β by human peripheral blood mononuclear cells (PBMCs) completes the antifungal armamentarium of C5a (75, 77).

Similarly, the anaphylatoxin C3a exerts a broad spectrum of antimicrobial effects including chemotaxis and immune cell activation (78). However, C3a-like peptides were also shown to act directly as antifungal weapons by binding to the *Candida* surface and inducing membrane perturbations (79).

In contrast, the influence of the terminal complement cascade on host defense against yeasts is less clear. In any case, the progress of the terminal pathway will lead to formation of the C5b-9 complex on the yeast cell surface. Although C5b-9 is also termed membrane attack complex (MAC), the thick fungal cell wall inhibits its lytic activity against fungal cells (unlike for gram-negative bacteria or erythrocytes) (3). However, deposited MAC may cause immune modulatory effects, as it induces a higher fungal mitochondrial activation and augments phagocytosis (80).

## Complement Evasion by *Candida*

### Masking

**Figure 3:**
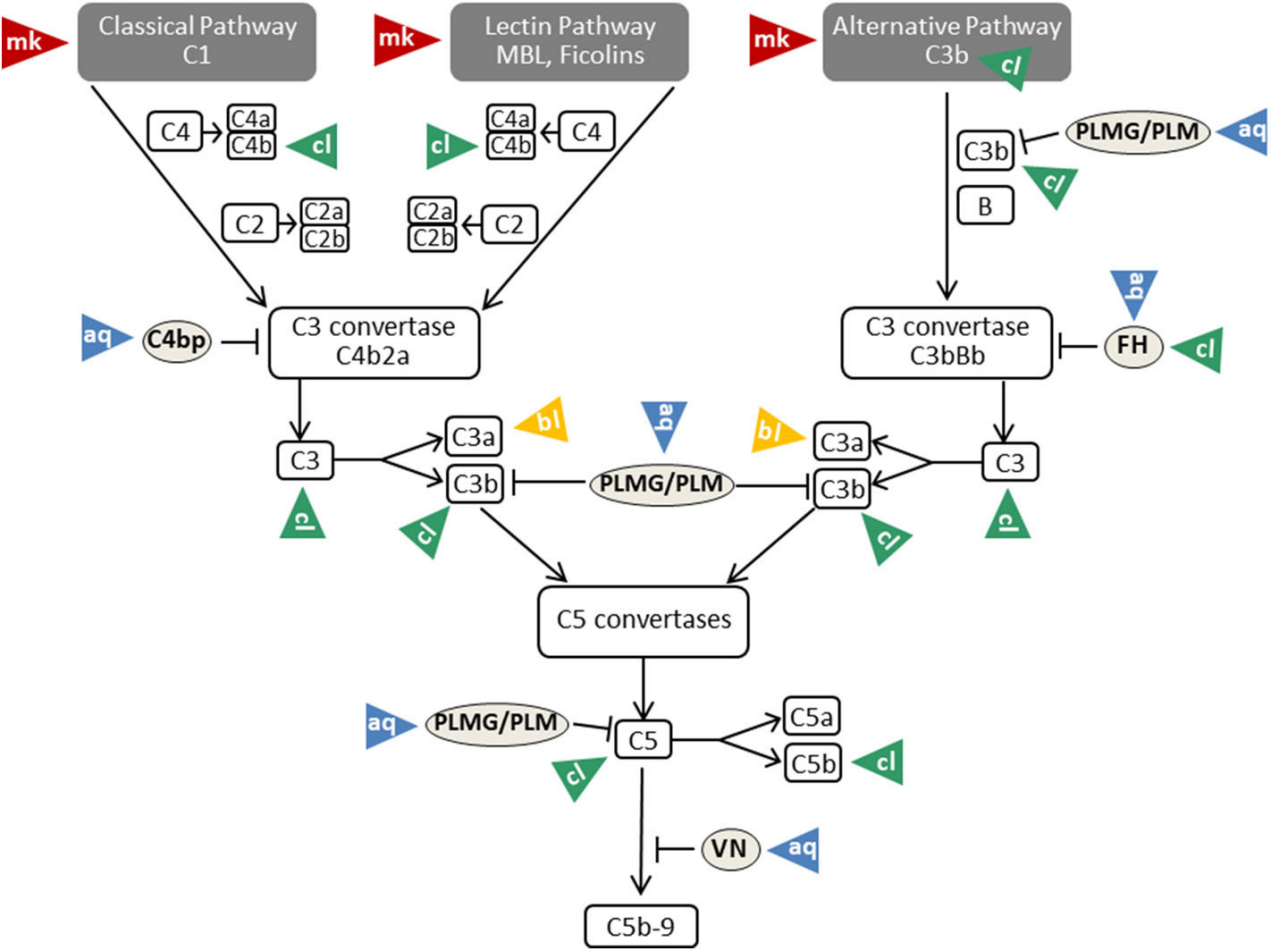
Mechanisms of complement evasion by *Candida*. The different evasion mechanisms affecting the complete flow of the complement cascade are indicated by triangles and labeled with mk (masking; red triangles), aq (acquisition; blue triangles), cl (cleavage; green triangles), and bl (blocking; yellow triangles). VN, vitronectin; PLMG, Plasminogen; PLM, Plasmin; FH, factor H. For further details and references, see text.

As described above, β-glucan molecules from the inner part of the *Candida* cell wall become exposed during infection or by treatment with the echinocandin caspofungin (34). Triggering of the alternative pathway of the complement system is a consequence of β-glucan exposure on the surface (35). Intact *Candida albicans* cells, however, are covered by a sheath of mannan that masks the complement-activating β-glucan layer (81) (Table 1). This mannan sheath provides resistance against alternative pathway activation (see Figure 3); conversion of mannose polysaccharides to polyalcohols or chemical removal of the surface mannan was shown to overcome this resistance (35). Specific antibodies against *C. albicans* mannan are a further tool to overcome this masking mechanism and to improve the outcome in experimental disseminated candidiasis (97). Interestingly, antibodies specifically recognizing β-mannans were protective against candidiasis, whereas α-mannan antibodies turned out to be non-protective. This difference perfectly correlated with the capacity of the antibodies to bind complement factor C3 on the yeast surface, a process that is much more efficient for the protective antibodies (97). The potency of mannan antibodies to overcome complement resistance is of particular interest, as most individuals have naturally occurring antibodies reactive with *Candida* mannan epitopes. However, these antibodies vary considerably regarding quantity and epitope specificity (98).

**Table 1:** Overview of the different types of complement evasion performed by *Candida* species and the underlying mechanisms. For further details, see text.

**Type of complement evasion**	**Mechanism**	***Candida* species**	**References**
Masking	Sheath of mannan covering β-glucan layer providing resistance against alternative pathway activation	*C. albicans*	(81)
Cleavage and blocking of complement proteins	Sap1-Sap3 degrade C3b, C4b, and C5 inhibiting opsonization by C3b and generation of anaphylatoxin C5a	*C. albicans*	(82)
	Sap2 cleaves FH, eliminating FH-mediated bridge between pathogen and neutrophils	*C. albicans*	(83, 84)
	Sap2 interferes with CR3 and CR4 expression on macrophages	*C. albicans*	(83)
	Sapp1 and Sapp2 cleave C3b, C4b and FH	*C. parapsilosis*	(85)
	Sapp2 degrades FHR5	*C. parapsilosis*	(85)
	Pra1 cleaves C3 at a unique site; the resulting C3a-like fragment has no effector functions and the C3b-like fragment is also not active and further degraded by FI and FH	*C. albicans*	(86)
	Pra1 blocks C3a derived from the action of C3 convertases	*C. albicans*	(86)
Recruitment of complement regulators	Acquisition of FH, FHL-1 by • Gpm1 • Pra1 • Hgt1 • Gpd2 – Competing with FB for C3b binding and inhibiting the assembly of the alternative pathway C3- and C5- convertases – Accelerating the decay of alternative pathway C3- and C5-convertases by displacing bound factor Bb – Enhancing FI-mediated cleavage and inactivation of C3b	*C. albicans*	(54, 72, 87–89)
	Acquisition of vitronectin by Gpm1 inhibiting C5b-7 insertion and C9 polymerization	*C. albicans*	(90, 91)
	Acquisition of C4BP by • Pra1 • Hgt1 – Acting as a cofactor of FI-mediated cleavage and inactivation of the soluble and cell-bound C4b and preventing the assembly of the C3 convertase C4b2b – Enhancing FI-mediated C3b degradation	*C. albicans*	(72, 92, 93)
	Acquisition of plasminogen by • Gpm1 • Gpd2 • Other proteins – Enhancing FI-mediated C3b degradation – Cleaving C3b and C5, when activated to plasmin	*C. albicans* *C. parapsilosis*	(54, 94–96)

### Cleavage and Blocking of Complement Proteins by *Candida*-Derived Proteases

*Candida albicans* produces a family of 10 related secreted aspartyl proteases (Sap proteins) that are considered to contribute substantially to pathogenicity by favoring adhesion, invasion, and tissue damage (99, 100). Furthermore, Sap proteins are of central relevance for pathogen survival in the host, since they destroy molecules of the immune system (antibodies, complement factors, cytokines) and thus limit microbicidal attacks (101). In this respect, elimination of an effective complement cascade as first-line defense is of special importance (Figure 3). Three members of the Sap family (Sap1-Sap3) were proven to bind and proteolytically degrade the complement proteins C3b, C4b, and C5 (82) and thereby subsequently inhibit complement activation (Table 1).

The functionality of this process was demonstrated by the findings that Sap proteins inhibit the opsonization of *C. albicans* by C3b and the generation of the anaphylatoxin C5a (82). A second complement-evading mechanism could be attributed to Sap2. It cleaves factor H (FH), a regulator of complement activity and, in addition, a putative bridging molecule between the pathogen and complement receptor CR3 (83) (Table 1). Losse et al. (84) revealed that FH, factor H-like protein (FHL-1) and factor H-related protein-1 (FHR-1) bind to *C. albicans* as well as to neutrophils. CR3 (CD11b/CD18) was identified to be the major receptor on neutrophils and thus facilitates the attachment of neutrophils to FH-bound *Candida*. When attached to *Candida* surface, FH and FHR1 enhanced the antimicrobial activity of the granulocytes and increased the killing of the pathogen (84). Secretion of Sap2 by *Candida* thus can contribute to immune evasion by proteolytically eliminating the FH-mediated bridge between pathogen and neutrophils. Furthermore, Sap2 interferes with CR3 and CR4 expression on macrophages (83) (Table 1).

The relevance of Sap proteins as virulence factors is not limited to *C. albicans*. A recent paper describes the functional characterization of Saps produced by *Candida parapsilosis* (85). *C. parapsilosis* possesses three secreted aspartyl protease-encoding genes (Sapp1-Sapp3). The purified enzymes Sapp1 and Sapp2 were demonstrated to efficiently cleave the complement components C4b, C3b, and FH; Sapp2 additionally degraded factor H-related protein 5 (FHR5) (Table 1). Macrophages phagocytosed and killed *C. parapsilosis* sapp1/2/3-/- deletion mutants more efficiently than wild type strains, indicating that Sap-mediated complement evasion is not limited to *C. albicans* (85). Two additional species that possess Sap genes, namely *C. tropicalis* (Sapt1-Sapt4) and *C. dubliniensis* (Sapcd1-Sapcd4, Sapcd7-Sapcd10), might also utilize Saps to modulate the complement system due to the high similarity of their Sap genes with those of *C. albicans* and *C. parapsilosis* (102, 103).

Saps are not the only *Candida*-derived proteases that contribute to virulence. The pH-regulated antigen 1 (Pra1) is described to interfere with complement-mediated antimicrobial functions by several mechanisms. Besides recruiting in its surface-bound form the complement inhibitors FH and C4BP to the *Candida* surface (see below), it is also released by *C. albicans* yeast and hyphal forms (87). Recombinant Pra1 was identified as a C3-degrading protease, cleaving C3 at a unique site (Table 1). Pra1 blocks effector functions of the resulting C3a fragment, whereas the Pra1-generated C3b-like fragment is processed and thus inactivated by the complement regulators factor I and factor H. Furthermore, Pra1 can also block C3a derived from the action of the C3 convertases (86) (Table 1).

### Recruitment of Complement Regulators FH and FHL-1 on the *Candida* Surface

Although factor H (FH) and factor H-like protein 1 (FHL-1), beside others, should prevent the detrimental effect of the complement system on endogenous cells, such proteins are frequently used by pathogens to avoid recognition or destruction by complement. Regarding FH and FHL-1, it is known so far that *Candida albicans* expresses four different so-called FH-binding proteins, namely phosphoglycerate mutase 1 (Gpm1), pH-regulated antigen 1 (Pra1), high affinity glucose transporter 1 (Hgt1), and glycerol-3-phosphate dehydrogenase 2 (Gpd2) (53, 54, 72, 88) (Table 1).

Gpm1 was the first protein shown to bind FH (54). The native protein has a mass of 27.5 kDa and regulates the catalytic conversion of 3-phosphoglycerate to 2-phosphoglycerate in glycolysis and the reverse reaction in gluconeogenesis in the cytoplasm (54, 90). Beside the localization in the cytoplasm, Gpm1 is present in the cell wall of yeast cells and hyphae, latter especially on the tip of the hyphae. The lack of classical signal and transport sequences implies that export from the cytoplasm to the yeast surface circumvents endoplasmic reticulum and Golgi, but rather occurs via a non-conventional transport mechanism (54). When located on the cell wall, Gpm1 binds different complement regulators, namely FH, FHL-1 as well as vitronectin and plasminogen (see below). Regarding FH, Gpm1 bears two binding sites for this protein, which are located at SCRs 6-7 and SCRs 19–20 (Figure 1), the former is shared with FHL-1. These regulators stay active when Gpm1 binds them to *Candida* (54). Even though this moonlighting protein was detected on the cell surface of different *Candida* species, namely *C. albicans, C. parapsilosis*, and *C. glabrata*, only little is known on the functions of Gpm1 in non-*albicans* species (104).

In 2009, 2 years after the first FH-binding molecule was described, Pra1 was discovered to bind FH to *Candida albicans*. In its monomeric form, Pra1 has a mass of 68 kDa and is composed of 299 amino acids (53, 105). It was initially described as a fibrinogen binding protein, which is localized on the cell wall of yeast and hyphae (105). The concentration is enhanced on the hyphal surface and notably high on the tips (53). Pra1 can either act as a pH-dependent zincophore, or manipulate complement to *Candida's* favor, as described above (53, 87, 106). Additionally, Pra1 binds directly or indirectly to different immune cells, resulting in contradictory effects. On the one hand, it can bind to CD4^+^ T-cells and inhibit cytokine secretion; on the other hand, it serves as a ligand for the integrin α_M_β_2_ (CD11b/CD18, CR3) expressed on neutrophilic granulocytes and therefore enables enhanced phagocytosis (107–109). FH is bound to Pra1 by two binding sites, namely SCRs 16–20, distinct for FH, and SCRs 5–7 (Figure 1), which is also a binding site for FHL-1. As the first four SCRs, mediating the complement regulator functions, are not affected by these different binding sites, pathogen-bound FH and FHL-1 stay functionally active (53).

In 2011, another FH-binding molecule named Hgt1 was discovered (72). This protein belongs to a large gene family of glucose transporters (20 proteins are described by now) and consists of 12 transmembrane domains, 545 amino acids, and weighs 60.67 kDa (110). Hgt1 can also be found in the cell wall of yeast cells and hyphae, where it can serve, in addition to its function as glucose transporter, as a binding molecule for the complement regulators FH and C4b-binding protein (C4BP) (72, 89). With binding of FH as main regulator of alternative pathway and of C4BP as main inhibitor of the classical and lectin pathway, Hgt1 has the capacity to comprehensively interfere with complement activation.

Gpd2, the fourth and by now last protein realized to bind FH, is made up of 371 amino acids, weighs 52 kDa and is localized on the surface of both yeast cells and hyphae (88). Initially, this protein was described as a nicotinamide adenine dinucleotide (NAD+)-dependent enzyme contributing to the degradation of glycerol (111). Gpd2 on *Candida* was shown to bind to human epithelial and endothelial cells, which might support *Candida* infection and dissemination in the host. In addition, Gpd2 controls the complement system by binding plasminogen via lysine residues (like Gpm1) and FH/FHL-1 by an amino acid sequence in SCR 7 (88) (Figure 1). FH/FHL-1 derived from individuals with a known human polymorphism on SCR7 (112, 113) may be unable to attach to Gpd2; it is interesting to speculate if these individuals show a better outcome in *Candida* infections.

Factor H binding by *Candida* might, however, also represent a double-edged sword for the pathogen. Since FH was described to also serve as a ligand for CR3 (114), *Candida*-bound FH might interact with CR3 on neutrophils and macrophages, thus bridging the pathogen to professional phagocytes and supporting clearance of the invader.

### Recruitment of Other Complement Regulators on the *Candida* Surface

The family of factor H, factor H-like proteins, and factor H-related proteins are not the only complement regulators that are exploited by *Candida* to evade from complement. The soluble plasma proteins C4BP, plasminogen and vitronectin were also described to attach to *Candida* surface, to retain their complement regulatory functions and to assist in complement evasion (19, 87, 115) (Figure 3).

Acquisition of vitronectin on the surfaces of pathogens is a common complement evasion mechanism that was described for bacterial and viral pathogens (116, 117). As mentioned above, vitronectin is an inhibitor of the terminal pathway of complement; by inhibiting the membrane insertion of C5b-7 and the polymerization of C9, it interferes with assembly of the C5b-9 complex and formation of a lytic pore [reviewed in (19, 118)]. Vitronectin was shown to be a ligand for the *Candida albicans* surface protein Gpm1 (90). Acquisition of vitronectin to *Candida* leaves its terminal pathway regulatory domain accessible, thus enabling inhibition of C5b-7 insertion and C9 polymerization (91) (Table 1). Furthermore, vitronectin also binds to *C. parapsilosis* and *C. tropicalis* pseudohyphae via various surface compounds (119), implying that a similar complement evasion mechanism might be possible for these *Candida* species.

To limit complement activation for survival in the presence of innate immunity, *Candida* exploits the efficacy of the main regulator of the classical and lectin pathway, C4BP. For this purpose, both the yeast and the hyphal forms of *Candida albicans* are capable of binding C4BP to the fungal surface (92, 120). Preferential binding sites are located on the tips of the germ tubes. The surface-attached C4BP retains its regulatory capacity, inactivates C4b and thus limits the flow of the complement cascade (92) (Table 1). The pH-regulated antigen 1 (Pra1) and high affinity glucose transporter 1 (Hgt1) of *Candida albicans* were described to be the corresponding C4BP-binding molecules that enable this mechanism of fungal complement evasion (72, 87). Pra1 expression levels of different clinical *Candida albicans* isolates correlated with C4BP binding activity, increased fungal virulence and enhanced survival of the pathogen in the presence of complement (93). The acquisition of C4BP on the fungal surface is not limited to *Candida albicans*. Meri et al. also proved the binding of this protein to the yeast form of *C. tropicalis, C. glabrata*, and *C. krusei*, but it is not known whether the bound C4BP remains regulatory active in these species (92).

The third usage of complement regulators for *Candida albicans* survival concerns plasminogen. *C. albicans* acquires plasminogen on its surface via attachment to Gpm1, and bound plasminogen can be converted into the active protease plasmin by urokinase-type plasminogen activator (54). A recent report even lists eight plasminogen-binding proteins on *Candida albicans* and four proteins in *Candida parapsilosis* (94, 95). As mentioned above, some reports describe plasminogen/plasmin as a trigger for complement activation with generation of active C5 fragments. However, various reports show opposing results and picture plasminogen as complement inhibitor. Barthel et al. mention plasminogen as a complement inhibitor that enhances FI-mediated C3b degradation and, activated to plasmin, cleaves C3b and C5 (96) (Table 1).

## An Emerging Field: *Candida auris* and Complement

In the last decade, *Candida auris* emerged as a multidrug-resistant yeast with severe cases of nosocomial infections. Outbreaks were observed all around the world in more than 30 countries (121, 122). The new species is intrinsically resistant to fluconazole and shows variable susceptibility to other azoles, echinocandins, and amphotericin B (122, 123). The genetic relation is closer to rarer *Candida* species such as *C. haemulonii* than to common species such as *C. albicans* and *C. glabrata* (123). Meanwhile, four distinct clades were distinguished with considerable inter-clade variation, implying independent emergence in multiple geographic regions.

The multidrug resistance strongly asks for deeper insights as a basis to develop immune-based supporting therapies. However, the knowledge about the interaction of *C. auris* with innate immunity is still limited. Xin et al. described that a mouse strain deficient in complement protein C5 (ΔC5) showed a high susceptibility for disseminated *C. auris* infection, compared to strains expressing C5. Immunosuppression with cyclophosphamide further substantially increased this susceptibility in ΔC5 mice, whereas treatment of animals with intact C5 gene had only minor effect. Detailed analysis of fungal burden in different organs revealed that expression of C5 is essential to limit the dissemination of *C. auris* into kidney, heart, and brain in infected mice (124).

Mimicry of complement receptors on the fungal surface is another link between *C. auris* and the complement system. The *Candida* complement receptor 3-related protein (CR3-RP) is a surface protein expressed by various *Candida* species during biofilm formation. CR3-RP is functionally and structurally related to the human CR3 (Mac-1, CD11b/CD18) expressed on neutrophils, monocytes, and macrophages. CR3-RP binds the human complement fragment iC3b and plays an important role in adherence to epithelial cells and in biofilm formation of different *Candida* species (125). Recent analysis confirmed the presence of CR3-RP also on the surface of *C. auris*. Blocking of CR3-RP with an antibody in the adherence phase inhibited *C. auris* biofilm formation. Furthermore, a polyclonal anti-CR3-RP antibody decreased the metabolic activity of pre-formed *C. auris* biofilms (126). Future work concerning *C. auris*-triggered complement activation, opsonization, and evasion mechanisms are necessary to get deeper insight into the interplay between this multidrug-resistant pathogen and the complement cascade.

## Conclusion and Outlook: Current and Future Aspects of *Candida*-Complement Research

As described in our review, complement as a fast-acting immune weapon with nearly overall presence in the body and a broad spectrum of pathogen recognition is of central relevance for local and invasive *Candida* infections.

Although comprehensive views on this interesting mutual interplay between a pathogen and the corresponding innate immune response already exist, some aspects ask for further studies. Only limited precise data are available on complement and local *Candida* lesions in mouth, gut, skin, and vagina. Since many complement proteins are acute-phase proteins, the extent of the topical synthesis of single complement proteins in different stages of disease would be interesting. Furthermore, the contribution of excessive complement activation to development and severity of local lesions is a relevant aspect to be clarified.

Another emphasis should be a broader insight into non-*albicans* species. Although some data are available on the expression of Saps or plasminogen-binding proteins for non-*albicans*, (e.g., for *C. parapsilosis* or *C. glabrata*, an overview about the relevance of single complement aspects for the different species is lacking). A special focus in this respect should be laid on *Candida auris*. The relevance of complement for the defense against this species is emphasized by the susceptibility of complement-deficient animals. However, it is unclear which activation pathway has the highest impact to trigger the cascade. The rather small number of patients makes it difficult to weight the role of MBL polymorphism for the susceptibility against *C. auris*. Furthermore, the evasion mechanisms of *C. auris* are still completely unknown. Here, the acquisition of factor H, a central evasion mechanism for other *Candida* species, deserves particular attention.

Exploitation of complement molecules by *Candida* to down-regulate inflammatory immune responses is another aspect that should be expanded to non-*albicans* species. *C. albicans* was described to secrete soluble β-glucan that binds to complement receptor CR3 on host monocytes. Consequently, monocytes form and release vesicles that transport TGF-β1. TGF-β1-transporting extracellular vesicles down-modulate inflammation in whole-blood cells and amplify the anti-inflammatory reaction of endothelial cells (127). This fascinating mechanism might also play a role for other *Candida* species. The battle between *Candida* and complement is old, but still reveals many new aspects.

## Author Contributions

VH wrote the section Recruitment of Complement Regulators FH and FHL-1 on the *Candida* Surface. GR wrote chapter AN EMERGING FIELD: *CANDIDA AURIS* AND COMPLEMENT and references. RW wrote section COMPLEMENT-MEDIATED ANTI-*CANDIDA* EFFECTS. CS wrote the other chapters. CL-F did proofreading and corrections. All authors contributed to the article and approved the submitted version.

## Conflict of Interest

The authors declare that the research was conducted in the absence of any commercial or financial relationships that could be construed as a potential conflict of interest.
